# Characteristic MRI Findings of upper Limb Muscle Involvement in Myotonic Dystrophy Type 1

**DOI:** 10.1371/journal.pone.0125051

**Published:** 2015-04-28

**Authors:** Kazuma Sugie, Miho Sugie, Toshio Taoka, Yasuyo Tonomura, Aya Kumazawa, Tesseki Izumi, Kimihiko Kichikawa, Satoshi Ueno

**Affiliations:** 1 Department of Neurology, Nara Medical University School of Medicine, Nara, Japan; 2 Department of Neurology, Nara Prefectural Rehabilitation Center, Nara, Japan; 3 Department of Radiology, Nara Medical University School of Medicine, Nara, Japan; Brighton and Sussex Medical School, UNITED KINGDOM

## Abstract

The objective of our study was to evaluate the relation between muscle MRI findings and upper limb weakness with grip myotonia in patients with myotonic dystrophy type 1 (DM1). Seventeen patients with DM1 were evaluated by manual muscle strength testing and muscle MRI of the upper limbs. Many DM1 patients presenting with decreased grasping power frequently showed high intensity signals in the flexor digitorum profundus (FDP) muscles on T1-weighted imaging. Patients presenting with upper limb weakness frequently also showed high intensity signals in the flexor pollicis longus, abductor pollicis longus, and extensor pollicis muscles. Disturbances of the distal muscles of the upper limbs were predominant in all DM1 patients. Some DM1 patients with a prolonged disease duration showed involvement of not only distal muscles but also proximal muscles in the upper limbs. Muscle involvement of the upper limbs on MRI strongly correlated positively with the disease duration or the numbers of CTG repeats. To our knowledge, this is the first study to provide a detailed description of the distribution and severity of affected muscles of the upper limbs on MRI in patients with DM1. We conclude that muscle MRI findings are very useful for identifying affected muscles and predicting the risk of muscle weakness in the upper limbs of DM1 patients.

## Introduction

Myotonic dystrophy type 1 (DM1) is the most common form of inherited myopathy in adults, presenting with myotonia and muscle weakness in the distal portion of the upper and lower limbs. DM1 patients also show general symptoms, including cataract, frontal baldness, endocrinological abnormalities such as hypogonadism or hyperinsulinemia associated with insulin resistance, and dementia or mental retardation [1–3]. Weakness of the distal portion of the upper limbs and grip myotonia are the earliest clinical signs of skeletal muscle involvement in DM1 patients and cause skilled movement disturbance. Therefore, management of upper limb muscles is very important in maintaining patients’ activities of daily living.

To our knowledge, muscle involvement on magnetic resonance imaging (MRI) of the upper limb muscles in DM1 has not been reported in detail previously. In this study, we evaluated the relation between upper limb muscle involvement and muscle MRI findings in DM1 patients.

## Patients and Methods

### Patients

Seventeen DM1 patients from 14 families (8 men and 9 women, mean age 51.1 years, standard deviation 11.8 years, range 30–68 years) were studied (Table 1). All patients showed evidence of degenerative myopathy on clinical examinations as well as myotonia on clinical or electromyographic examinations or both. All patients gave fully informed consent before participation. Neurologically, the patients were examined and evaluated by at least three neurologists. Muscle strength was graded according to the Modified Medical Research Council (MRC) scale, which classifies muscle weakness in ten degrees from normal strength (10) to no voluntary movement (0) [4]. The definition of grip myotonia in this study was as follows: following a forceful grip, there is a delayed ability to relax the grip. The clinical features of the patients are summarized in Table 1.

**Table 1:** Clinical characteristics of 17 patients with myotonic dystrophy type 1.

Pt	Age/Sex	Grip myotonia (Rt/Lt)	Grasping Power (Kg)	CTG repeats	Duration (years)	Wrist flexion	Wrist extension	Elbow flexion	Elbow extension
1	62/F	+/+	5/7	100	7	7/7	8/8	10/10	9/9
2	59/M	+/+	8/6	100	24	8/8	8/8	10/10	10/10
3	55/M	+/+	15/15	100	16	9/8	9/9	10/10	10/8
4	57/M	+/+	9/8	100	12	6/6	6/6	7/7	8/8
5	34/M	+/+	12/12	200	5	8/8	10/10	10/10	10/10
6	45/M	+/+	5/7	500	12	8/8	8/8	10/10	10/10
7	36/F	+/+	3/2	500	9	8/8	8/8	9/8	5/6
8	47/F	+/+	9/8	600	7	8/8	8/6	10/10	8/8
9	59/M	+/+	0/0	700	19	6/6	7/7	8/8	6/6
10	36/F	+/+	8/7	800	7	8/8	8/8	10/10	10/10
11	38/M	+/+	7/7	800	12	8/8	8/8	10/10	8/8
12	62/F	+/+	0/0	1000	15	6/6	7/7	8/8	8/8
13	68/M	+/+	0/0	1000	33	1/1	1/1	1/1	1/1
14	30/F	+/+	7/7	1100	8	4/4	4/4	10/10	4/4
15	64/F	+/+	7/7	1500	30	8/8	9/9	8/8	10/10
16	59/F	+/+	0/0	1800	36	8/7	8/8	8/8	8/6
17	57/F	+/+	0/0	2300	33	5/5	5/5	5/5	5/5

This study was approved by the Ethics Committee of the Nara Medical University School of Medicine. All the involved subjects gave their written informed consent.

### Genetic Studies

Genetic studies of the expansion of CTG repeat length in the 3’ untranslated lesions of myotonin protein kinase were performed with the use of DNA separated from leukocytes. Digestion with Eco RI and Bgl I restriction enzymes, separation of DNA fragment by gel electrophoresis, transfer of the DNA fragment to a nylon membrane by Southern blotting, hybridization with radiolabeled DM1-specific probe, and finally visualization by autoradiography were performed [5].

### Muscle MRI

All MRI examinations were performed using a 1.5 Tesla superconducting magnet (GE-Sigma, 1.5 Tesla). The upper limb was examined in 5-mm axial slices using T1- (TR 500/TE 15ms) and T2- (TR 2000/TE 22–90ms) weighted SE sequences in all DM1 patients. For all sequences, the same geometric parameters were used: field-of-view 140 mm, matrix size 197 × 256. The average total scan time was 30 minutes per patient. A phased array coil was used as the receiver coil. The left upper limb of all patients underwent MRI scanning. Muscle MRI findings were evaluated by two neurologists (MS and KS) and a radiologist (TT).

We analyzed individual muscles and subsequently categorized the overall degree of upper limb involvement. The following individual muscles were assessed unilaterally: biceps brachii (BB), triceps brachii (TB), and brachialis (B) muscles in the upper arm; and brachioradialis (BR), flexor carpi ulnaris (FCU), flexor carpi radialis (FCR), flexor digitorum profundus (FDP), flexor digitorum superficialis (FDS), flexor pollicis longus (FPL), abductor pollicis longus (APL), extensor pollicis longus and brevis (EP), extensor carpi ulnaris (ECU), extensor carpi radialis (ECR), extensor digitorum communis (EDC), pronator teres (P), and supinator (S) in the forearm. These 16 muscles were assessed on T1-weighted sequences for the presence of fatty infiltration, evaluated according to Fischer’s semi-quantitative scale [6]: 0—normal appearance, 1—occasional scattered T1 hyperintensity, 2—confluent areas of T1 hyperintensity <50% of muscle involved, 3—confluent areas of T1 hyperintensity >50% of muscle involved, and 4—complete replacement of muscle with fat. The score of 0 to 4 for each muscle was changed to—to ++++ in Table 2.

**Table 2:** Distribution of affected muscles detected by muscle MRI.

Pt	BB	TB	B	BR	FCU	FCR	FDP	FDS	ECU	ECR	EDC	S	P	APL	EP	FPL	MRI fat score
1	-	-	-	-	-	-	+	-	-	-	-	-	-	+	+	-	0.19
2	-	-	-	-	-	-	+	+	-	-	-	-	-	+	+	-	0.25
3	-	+	-	-	-	-	+	-	-	-	-	-	-	-	+	-	0.19
4	-	-	-	-	-	-	+	-	-	-	-	-	-	-	-	-	0.06
5	-	-	-	-	-	-	+	-	-	-	-	-	+	-	-	+	0.19
6	+	+	+	-	-	-	+	+	-	-	-	+	-	+	+	+	0.56
7	+	+++	+	-	-	-	++	+	-	-	-	+	+	+	+	-	0.75
8	+	-	+	-	-	-	+	-	-	-	-	-	-	-	-	-	0.19
9	-	+	-	-	-	-	++	+	-	-	-	+	-	+	+	+	0.5
10	-	-	-	-	-	-	+	-	-	-	-	-	-	+	+	+	0.25
11	-	-	-	-	-	-	+	-	-	-	-	-	-	+	+	+	0.25
12	+	+	+	-	+	-	++	+	-	-	-	-	-	+	+	+	0.63
13	++	+++	++	+	+	+	++	+	+	+	+	+	+	+	+	+	1.31
14	-	-	-	-	-	-	+	+	-	-	-	-	-	+	-	-	0.19
15	+	+	+	-	-	-	++	+	-	-	-	-	-	+	+	+	0.56
16	+	++	+	-	-	+	+++	+	-	-	-	+	-	+	+	+	0.81
17	+	++	+	+	+	+	+++	+	-	-	+	+	+	+	+	+	1.06

BB = biceps brachii; TB = triceps brachii; B = brachialis; BR = brachioradialis; FCU = flexor carpi ulnaris; FCR = flexor carpi radialis; FDP = flexor digitorum profundus; FDS = flexor digitorum superficialis, FPL = flexor pollicis longus; APL = abductor pollicis longus; EP = extensor pollicis longus and brevis; ECU = extensor carpi ulnaris; ECR = extensor carpi radialis; EDC = extensor digitorum communis; P = pronator teres; S = supinator.

- = normal; + mild change; ++ = mild to moderate change; +++ = moderate change; ++++ = severe change.

### Statistical analysis

An overall MRI involvement score was established for each patient by calculating the mean of the quantitative scores of all 16 muscles. We used the MRC scale on the left side, which corresponded to the side examined by muscle MRI. Statistical analysis was performed with the use of Statcel 3 (OMS, Inc., Tokorozawa, Saitama, Japan) to assess correlations between the overall MRI involvement score and clinical symptoms or the numbers of CTG repeat lengths by Spearman’s rank correlation coefficient. P values of <0.05 were considered to indicate statistical significance.

## Results

**Fig 1:**
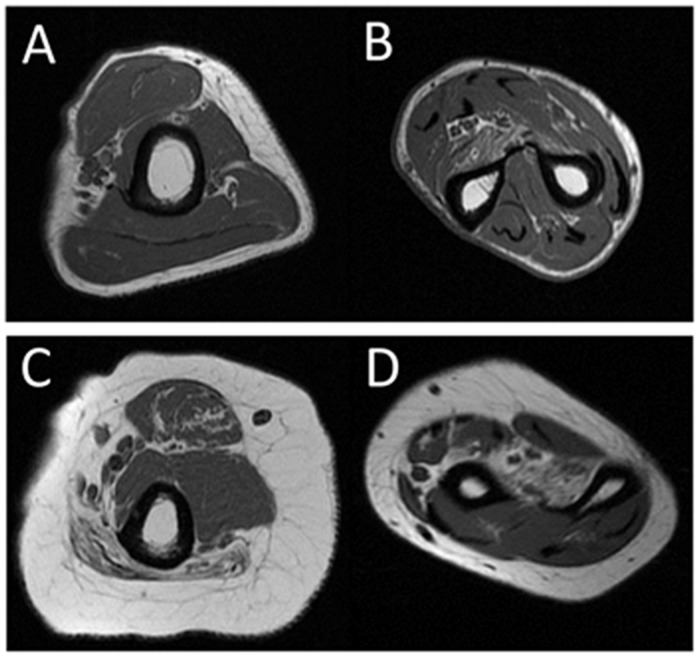
Muscle MRI findings of the upper limb muscles in myotonic dystrophy type 1. Typical findings of muscle MRI in a patient with mild disease, patient 4 (A, B), and a patient with severe disease, patient 7 (C, D), as summarized in Table 1. On T1-weighted images, only the FDP muscle showed high intensity signals with fatty degeneration in patient 4. In patient 7, the FDP muscle as well as a few other muscles showed high intensity signals with fatty degeneration. A, C = arm muscles; B, D = forearm muscles.

**Fig 2:**
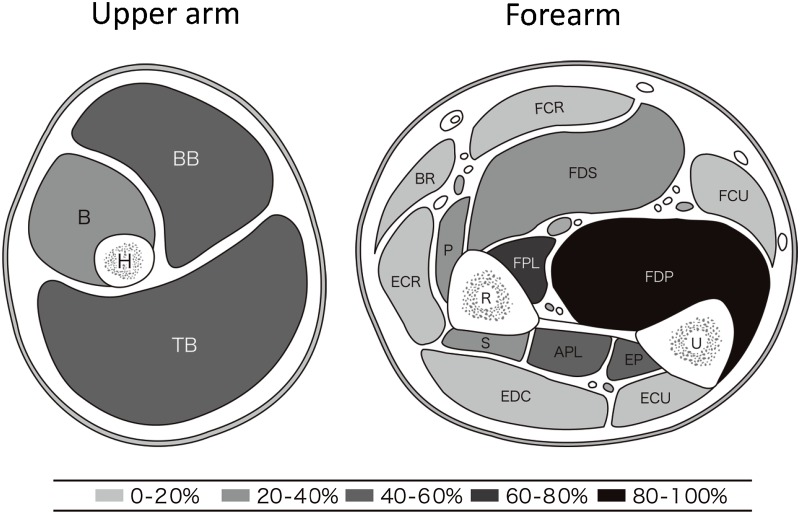
Frequency of muscles in the upper limbs showing >30% fat infiltration. BB = biceps brachii; TB = triceps brachii; B = brachialis; BR = brachioradialis; FCU = flexor carpi ulnaris; FCR = flexor carpi radialis; FDP = flexor digitorum profundus; FDS = flexor digitorum superficialis, FPL = flexor pollicis longus; APL = abductor pollicis longus; EP = extensor pollicis longus and brevis; ECU = extensor carpi ulnaris; ECR = extensor carpi radialis; EDC = extensor digitorum communis; P = pronator teres; S = supinator.

Clinical features of all DM1 patients are summarized in Table 1. Typical muscle MRI findings in a patient with mild disease (patient 4) and a patient with severe disease (patient 7) are shown in Fig 1. The distribution of affected muscles as detected by muscle MRI is shown for all patients in Table 2. All DM1 patients showed T1-weighted high intensity signals for the presence of fatty infiltration in the FDP muscle only (p<0.001). Patients with severe weakness showed high intensity signals also in the FPL, APL, and EP muscles at the forearm level, as well as the BB and TB muscles at the arm level. Patients with more advanced disease also showed involvement of the P, S, and B muscles. The distribution of affected muscles is shown in Fig 2. In general, the FDP, FPL, APL, and EP muscles were severely affected. However, the flexor carpi, extensor carpi, and EDC muscles were relatively spared, even in patients with advanced disease (Fig 2).

**Fig 3:**
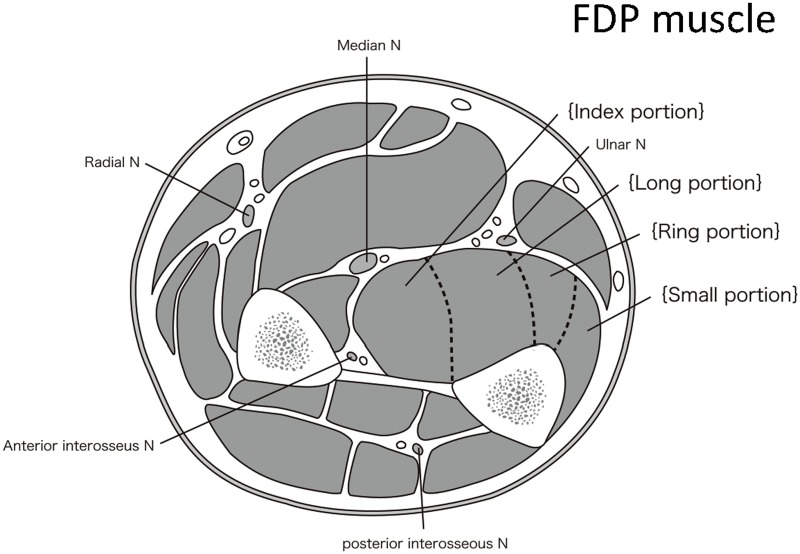
Anatomic transverse section of the forearm muscles. The FDP muscle, the most affected in all DM1 patients, contributes to flexion of the four fingers from the index finger to the small finger. In our study, the index finger and the long portions of the FDP muscle were most affected. These areas are surrounded by the median, ulnar, and posterior interosseous nerves and the ulnar bone.

The FDP muscle, the most affected in all DM1 patients, contributes to flexion of four fingers from the index finger to the small finger. In our study, the index finger and the long portions of the FDP muscle were most affected (Fig 3).

**Fig 4:**
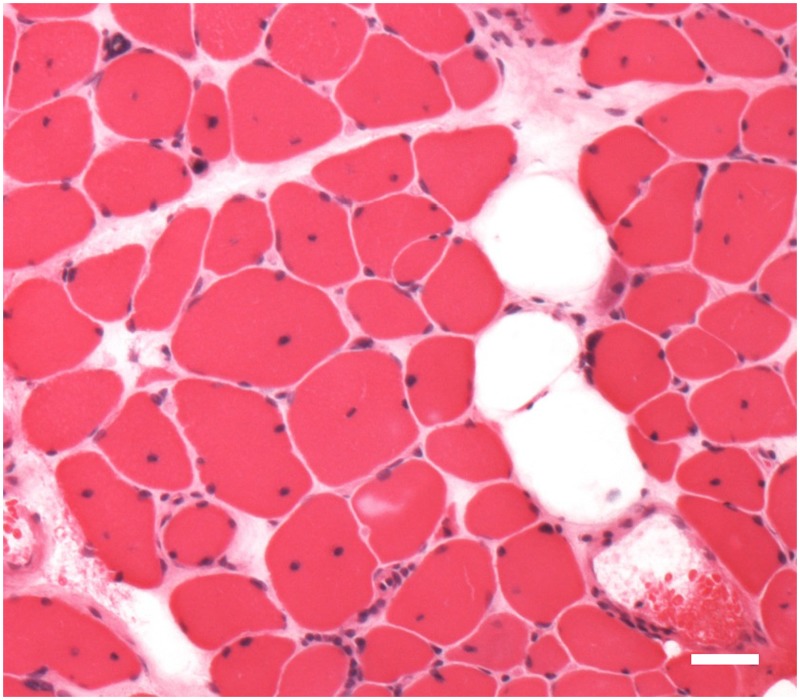
Histopathological findings of a biopsy specimen of the left biceps brachii muscle (patient 7 in Table 1). Histopathological examination of a biopsy specimen showed characteristic pathological findings, including central nuclei, variable ranges of fiber size, and fatty degeneration. H&E stain, *Bar* = 30 μm.

The correlations between the muscle MRI findings and the histopathological findings of biopsy specimens of the muscle were examined in patients 4, 7, and 15. In patients 7 and 15, T1-weighted high-intensity signals on MRI of the biceps muscle were associated with characteristic pathological findings, such as central nuclei, variable ranges of fiber size, and fatty degeneration (Fig 4). In contrast, the biceps muscle in patient 4, associated with no high-intensity signals on MRI, showed mild pathological findings without fatty degeneration (data not shown).

**Fig 5:**
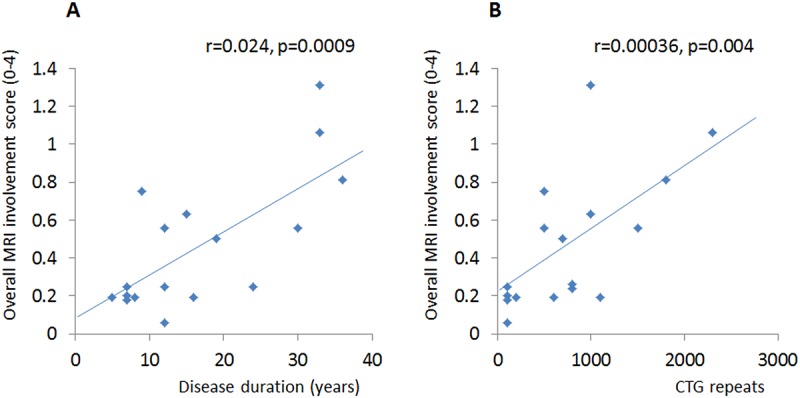
Correlations of fatty infiltration on MRI with disease duration (A) and the numbers of CTG repeats (B). Scatter-plots of overall MRI fat score versus disease duration (**A**) or the numbers of CTG repeats (**B**) in all patients. Significant correlations were observed in both **A** (r = 0.024, p = 0.0009) and **B** (r = 0.00036, p = 0.004).

The correlations between the MRC scale and MRI involvement score were significant in both the upper arm (p <0.05) and the forearm (p <0.05). The overall MRI involvement score of the upper limb muscles significantly correlated with the disease duration (r = 0.024, p = 0.0009) and the numbers of CTG repeats (r = 0.00036, p = 0.004), despite the diversity in phenotype and genotype (Fig 5). There was no significant correlation between the overall MRI involvement score and age. However, there was a trend toward a higher MRI involvement score in older patients (r = 0.012, p = 0.11).

## Discussion

Muscle MRI has been reported to be the most important imaging tool for the *in vivo* assessment of patients with various myogenic disorders, such as inclusion body myositis [7], polymyositis [8], and Duchenne muscular dystrophy [9]. Several studies have demonstrated lower limb muscle involvement in DM1 patients [10–13]; however, the detailed distribution of upper limb muscle involvement has not been reported previously.

To our knowledge, this is the first study to report the detailed distribution and severity of affected upper limb muscles on MRI in patients with DM1. Our results showed that all DM1 patients who presented with very mild to severe upper limb weakness showed T1-weighted high intensity signals in only the FDP muscles. In fact, FDP muscle involvement was already evident in early-stage disease. The FDP muscle fans out into four tendons (one to each of the index to small fingers) attached to the palmar base of the distal phalanx. We demonstrated that the index finger and the long portion of the FDP muscle were particularly affected, even in early disease. This area has anatomical specificity and is the deepest region surrounded by the median, ulnar, and posterior interosseous nerves and the ulnar bone. However, the reason for the earliest damage in this region remains unclear.

In addition, our results showed that the FDP muscle was most affected, followed by the APL and EPL muscles (Fig 1). These three muscles had more specific involvement and were located in deeper positions. Patients with severe weakness, moreover, showed high T1-weighted signal intensity extending to the FDP, FPL, APL, and EP muscles at the forearm level, as well as the BB and TB muscles at the arm level. In contrast, the peripheral muscles of the forearm were relatively preserved.

There is no satisfactory explanation for the distribution of muscular changes associated with different dystrophic processes. As one possible factor, a previous study suggested that anatomical characteristics such as shape, length, or extension over one or two joints might be contributing factors affecting muscle strength [14]. Another possible factor is the distribution of the two histopathological types of muscular fibers, type 1 and type 2, [15], as well as the selective alteration of these fiber types in some diseases, such as atrophy of type 1 fibers in DM1.

Histopathological findings of biopsy specimens of the muscle were associated with T1-weighted high-intensity signals on MRI findings in all three patients who underwent muscle biopsy in our study. MRI findings may thus be useful for estimating the underlying pathological changes of involved muscles. However, further studies of larger groups of patients are necessary to establish the correlation between histopathological findings and MRI findings.

Our study showed that the MRC scale correlated with the MRI involvement score in both the upper arm and forearm. Interestingly, the correlations of MRI muscle involvement with disease duration and the number of CTG repeats were also strongly positive. In fact, a recent study has shown that muscle weakness of wrists and elbows significantly correlate with the number of CTG repeats [16]. This finding may support the positive correlations of MRI muscle involvement with the number of CTG repeats and the MRC scale in our study. Our results also showed a trend toward a positive correlation between the MRI involvement score and age, although statistical significance was not reached. These findings may reflect the chronic progressive nature of DM1. Therefore, information obtained by muscle MRI may contribute to the estimation of prognosis.

Grip myotonia is often a prominent symptom in DM1 patients. However, because loss in power at the wrist is noted even in early disease, permanent distal muscle weakness and grip myotonia are the earliest clinical signs of skeletal muscle involvement. On the other hand, recessive myotonia congenita (RMC) is an autosomal recessive, inherited generalized myotonia without permanent muscle weakness. RMC is caused by muscle chloride channel dysfunction, similar to DM1. A previous study of RMC reported that myotonia and transient weakness alone are not associated with skeletal muscle signal changes on MRI [17]. Therefore, the muscle involvement seen on MRI in DM1 patients may not result from chloride channel dysfunction, but is clearly a consequence of the chronic dystrophic disease process.

In conclusion, muscle MRI findings are very useful for estimating the distribution and severity of affected muscles and anticipating muscle weakness in the upper limbs of DM1 patients. Our results showed that muscle involvement of the upper limbs on MRI strongly correlated positively with the disease duration and the numbers of CTG repeats. Therefore, information obtained by muscle MRI can contribute to the estimation of prognosis.
